# Autosomal Recessive Stickler Syndrome

**DOI:** 10.3390/genes13071135

**Published:** 2022-06-24

**Authors:** Thomas R. W. Nixon, Allan J. Richards, Howard Martin, Philip Alexander, Martin P. Snead

**Affiliations:** 1 Vitreoretinal Research Group, John van Geest Centre for Brain Repair, University of Cambridge, Forvie Site, Robinson Way, Cambridge CB2 0PY, UK; trwn2@cam.ac.uk (T.R.W.N.); ar204@medschl.cam.ac.uk (A.J.R.); hm208@cam.ac.uk (H.M.); 2 Vitreoretinal Service, Addenbrooke’s Hospital, Cambridge University Hospitals NHS Foundation Trust, Hills Road, Cambridge CB2 0QQ, UK; philip.alexander@addenbrookes.nhs.uk

**Keywords:** stickler syndrome, *COL9A1*, *COL9A2*, *COL9A3*, *COL11A1*, *LOXL3*, *LRP2*, retinal detachment, collagen

## Abstract

Stickler syndrome (SS) is a genetic disorder with manifestations in the eye, ear, joints, face and palate. Usually inherited in a dominant fashion due to heterozygous pathogenic variants in the collagen genes *COL2A1* and *COL11A1*, it can rarely be inherited in a recessive fashion from variants in *COL9A1*, *COL9A2*, and *COL9A3*, *COL11A1*, as well as the non-collagen genes *LRP2*, *LOXL3* and *GZF1*. We review the published cases of recessive SS, which comprise 40 patients from 23 families. Both homozygous and compound heterozygous pathogenic variants are found. High myopia is near-universal, and sensorineural hearing loss is very common in patients with variants in genes for type IX or XI collagen, although hearing appears spared in the *LRP2* and *LOXL3* patients and is variable in *GZF1*. Cleft palate is associated with type XI collagen variants, as well as the non-collagen genes, but is so far unreported with type IX collagen variants. Retinal detachment has occurred in 18% of all cases, and joint pain in 15%. However, the mean age of this cohort is 11 years old, so the lifetime incidence of both problems may be underestimated. This paper reinforces the importance of screening for SS in congenital sensorineural hearing loss, particularly when associated with myopia, and the need to warn patients and parents of the warning signs of retinal detachment, with regular ophthalmic review.

## 1. Introduction

Stickler syndrome (SS) is a condition that affects the eye (congenital myopia, retinal detachment, lamellar cataract, perivascular lattice degeneration), ear (both conductive and high-frequency sensorineural hearing loss), joints (hypermobility and premature arthritis), face and palate (mid face hypoplasia, Pierre Robin sequence, cleft palate) [1]. It is the most common cause of familial retinal detachment, and the most common cause of childhood retinal detachment. SS is caused by genetic variants in genes encoding, or affecting the assembly of, types II, IX and XI collagen, and can be inherited in both dominant (common) and recessive (rare) modes of inheritance.

The most common form is Type 1 SS, caused by heterozygous loss-of-function variants of *COL2A1* causing haploinsufficiency [2]. These patients have a very high risk of retinal detachment, above 50% lifetime risk, and if they have retinal detachment in one eye, have above an 80% chance of retinal detachment in the fellow eye [3]. As a significant proportion of these retinal detachments are due to giant retinal tears at the oro-retinal junction, prophylactic cryoretinopexy at this location has been shown to be highly effective at significantly reducing the risk of retinal detachment [3,4]. They usually have high myopia and a characteristic membranous vitreous anomaly [5]. Hearing is affected in half of patients with Type 1 SS [6]. In a combined group of Type 1 and Type 2 SS (the dominant forms), joint pain is reported in 41% of patients before age 10 and 90% of patients over 40 years of age [1], and palate anomalies are described in 45% of Type 1 SS and 28% of Type 2 SS patients [7].

Type 2 SS is caused by heterozygous dominant negative variants in *COL11A1* [8]. Retinal detachment is still a significant feature, reported in 42% of cases, although less commonly due to giant retinal tears [9]. They usually have high myopia and exhibit a different vitreous phenotype [5]. Hearing is more commonly affected, in 69–83% [6,10].

There was an entity known as ‘non-ocular Stickler Syndrome’ due to variants in *COL11A2*, but this has been reclassified as oto-spondyl-megaepiphyseal dysplasia.

Recessive SS has been reported less frequently than dominant SS, but has now been described in 40 patients from 23 families [11,12,13,14,15,16,17,18,19,20,21,22,23,24,25,26]. Both homozygous and compound heterozygous inheritance patterns have been described. Most commonly affected are the three genes for type IX collagen, *COL9A1*, *COL9A2* and *COL9A3*. Recessive *COL11A1* loss-of-function variants have previously been felt to be severely life-limiting, causing fibrochondrogenesis [27,28], but there are now several cases described of a Stickler phenotype, where the variants affect exon 9, which is alternatively spliced and not included in mature chondrocytes, resulting in SS with severe hearing loss. Stickler-like phenotypes have also been described in patients with loss of function variants in *LOXL3*, which encodes an enzyme that crosslinks type II collagen and *LRP2* and *GZF1*, for which the biological mechanism is less clear.

In this paper, we review the current published cases of recessive SS to give an up-to-date description of the phenotype of recessive SS in comparison with dominant SS, and highlight that compound and apparently benign heterozygous variants can be an important consideration in the genetic analysis of patients with clinical features of SS.

## 2. Recessive Stickler Variants in Genes for Type IX Collagen

The most common forms of recessive SS result from variants affecting genes *COL9A1*, *COL9A2* and *COL9A3* [11,12,13,14,15,16,17,18]. Table 1, Table 2 and Table 3 show the reported features of eight patients from four families with *COL9A1* variants, seven patients from four families with *COL9A2* variants and seven patients from four families with *COL9A3* variants. All are homozygous variants except one patient with a compound heterozygous pattern, with each allele possessing a different variant, causing a premature stop codon. It is notable that all patients have sensorineural hearing loss, mostly reported as moderate to severe. All patients are myopic, mostly high myopia (>−6D), and most have abnormal vitreous, usually hypoplastic. One patient from each variant group has had retinal detachment, giving a rate of 13.6% (this includes patient 11-II from our National Stickler Service who had not had retinal detachment at the time of publication, but has now suffered unilateral retinal detachment, age 24). Joint pain was a feature in 22.7% of patients. Facial features were possibly present in 40.9%, but cleft palate was not reported in any of these patients.

**Figure 1:**
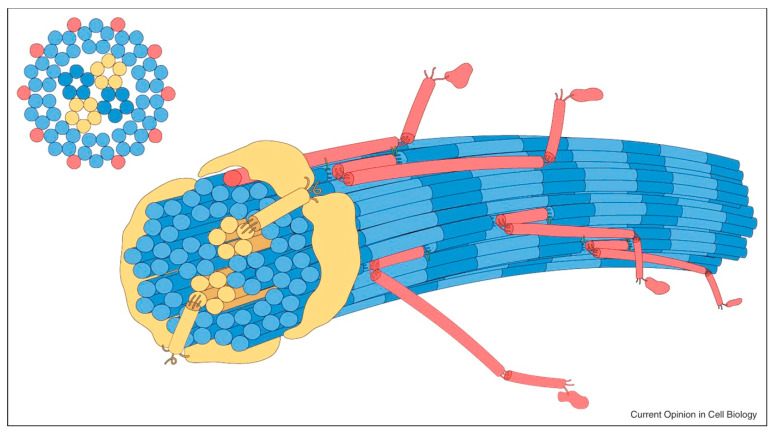
Schematic of the microfilament composition of collagen in cartilage. Blue: collagen II molecules; yellow: collagen XI molecules; red: collagen IX molecules. Collagen IX molecules project outwards and interact with the local environment. Adapted from [29].

The genes *COL9A1*, *COL9A2* and *COL9A3* encode polypeptide chains α1, α2 and α3, respectively, which co-assemble to form the heterotrimer of type IX collagen [29]. These fibres are then assembled onto the outside of the collagen II/XI fibril (see Figure 1) with covalent links to type II collagen as well as other type IX collagens, suggesting that they may form a macromolecular bridge between collagen fibrils, as well as other matrix constituents [30]. It is interesting to note that biallelic loss of function of *COL2A1* is lethal, and biallelic loss of function of *COL11A1* gives the severe condition of fibrochondrogenesis [27,28] unless the variant is alternatively spliced out of the collagen in mature chondrocytes, but biallelic loss of any one of the α chains for type IX collagen results in the milder skeletal SS phenotype. This may suggest that the remaining two α chains are able to form an alternative heterotrimer, which can fulfil some, but not all, of the functions of the normal heterotrimer. There is a report of two families with a heterozygous *COL9A3* variant segregating with a Stickler phenotype, which may represent a dominant negative effect on the heterotrimer [31].

## 3. Recessive Stickler Variants in Genes for Type XI Collagen

*COL11A1* normally results in type 2 SS via heterozygous dominant-negative variants, but several families have been reported with biallelic loss of function variants. Table 4 shows the reported features of seven patients from six families with biallelic *COL11A1* variants, of which two families are affected by homozygous variants and four families are affected by compound heterozygous variants [19,20,21,22]. All patients have sensorineural hearing loss, mostly severe to profound. Most patients have high myopia, although notably, one patient has mild hypermetropia. Retinal detachment is reported in one patient (14%), but retinal tears in a second (14%). No patients reported joint pain. Two patients (28%) had Pierre Robin sequence and a further two patients (28%) had cleft palate.

Variants in *COL11A1* causing Type 2 SS are usually heterozygous dominant-negative variants, with an abnormal α1 chain disrupting the function of the other α chains, resulting in a dysfunctional protein [8]. Heterozygous loss of function does not cause SS, although there is a suggestion it may be associated with myopia and early-onset hearing loss [28]. Homozygous loss-of-function variants have been reported to cause the severe skeletal dysplasia fibrochondrogenesis, which is usually neonatally lethal, although there are reported nonlethal cases [27,28].

In cases of recessive SS due to biallelic *COL11A1* variants, with the exception of patient 16-I, all the cases are ‘rescued’ from fibrochondrogenesis by the location of the variant on one or both alleles affecting the alternatively spliced exon 9. While exon 9 is expressed in both vitreous and immature chondrocytes, it is not expressed in mature chondrocytes [32], apart from in Meckel’s cartilage [33], which forms the malleus and incus of the inner ear, as well as the anterior ligament of the malleus tympanic plate [34]. This may explain why there is a more severe hearing than skeletal phenotype in these patients. In tissues where it is not expressed, the variant would be naturally removed from transcripts in those tissues and therefore have no effect. These exon 9-affecting variants are either homozygous or paired with another *COL11A1* variant, which either causes loss of function, or itself may be pathogenic with a dominant negative effect, which is then made more severe by the effect of the variant in exon 9-expressing tissues. The sparing of mature chondrocytes from the effect of these variants explains the SS phenotype, albeit often with severe hearing loss, instead of the severe skeletal dysplasia fibrochondrogenesis (see above).

Intriguingly, in patient 16-I, the c.2607A > G, p.Ala869Ala ‘silent’ variant was shown to cause splicing errors, with both normal and abnormal transcripts produced, resulting in approximately half the normal level of functional transcripts. This variant was paired with c.5398G > T, p.Gly1800Cys, which would be predicted to be pathogenic due to its effect on the pattern of cysteines in the C-propeptide. This case highlights the importance of functional analysis of apparently benign variants inherited in tandem with known pathogenic variants on the other allele.

## 4. Recessive Stickler Variants in Non-Collagen Genes

Three non-collagen genes have been identified to cause an SS-like phenotype: *LRP2, LOXL3* and *GZF1*. Table 5 shows the reported features of 11 patients from five families with variants in one of these genes [23,24,25,26]. Hearing was normal with the *LRP2*- and *LOXL3*-variant families, but there was hearing loss, uncharacterised, in two of the five patients with the *GZF1* variant. Three patients have had retinal detachment (27%), all before the age of 20, with one due to a giant retinal tear, classical for SS. All patients are myopic, and where the vitreous is reported, it is abnormal. One patient complained of joint pain, and one has severe kyphoscoliosis with compromised lung function. Four (36%) patients have facial involvement and two (18%) have cleft palate.

*LRP2* loss-of-function variants are known to cause Donnai–Barrow syndrome (DBS) or facio-oculo-acoustico-renal (FOAR) syndrome [23]. This has some SS-like features, with myopia and sensorineural hearing loss, and separate features including hypertelorism, prominent brow, enlarged fontanelle, agenesis of the corpus callosum and developmental delay. In family 19, the absence of agenesis of the corpus callosum, developmental delay or diaphragmatic hernia, and the presence of abnormal vitreous, giant retinal tear and joint pain are the features that are phenotypically more consistent with SS. *LRP2* encodes lipoprotein receptor-related protein-2, an endocytic transmembrane receptor. A direct molecular interaction with collagen is not described.

*LOXL3* encodes lysyl oxidase-like 3, an enzyme involved in crosslinking collagen chains including type II collagen. Loss-of-function *LOXL3* variants have also been associated with early-onset myopia [35]. The patients in this case have been described as having cleft palate with flat midface and micrognathia in two cases, and premature vitreous separation in another case, which both suggest a degree of pathology more than myopia alone.

*GZF1* encodes GDNF-inducible zinc finger protein 1, a transcription factor [26]. The five patients in this case all have high myopia, two have retinal detachment, all have joint hypermobility, two with multiple joint dislocations, and one has severe kyphoscoliosis with compromised lung function. Only two patients are reported to have hearing loss, but it is not reported whether any formal audiological assessment took place.

## 5. Discussion

Recessive SS is an uncommonly reported, but important condition. Table 6 compares the features of recessive SS with dominant SS. In cases of clinically likely Stickler Syndrome but without *COL2A1* or *COL11A1* pathogenic variants detected, recessive SS must be considered, particularly in de novo cases and, again, highlighting the importance of the functional analysis of apparently benign variants inherited in tandem with known pathogenic variants on the other allele. Congenital sensorineural hearing loss is a consistent feature, and SS is a diagnosis that must be considered in these patients so that screening for ophthalmological problems can also occur, especially as dual-sensory impairment can have huge implications for development. We do not know the final lifetime risk of retinal detachment in recessive Stickler Syndrome, but it already appears higher than the general population, and these patients must be advised on the warning symptoms of posterior vitreous detachment and retinal detachment so they can seek timely assessment and treatment, and probably warrant long term follow-up. It is also important to note the young age of the patients in all of these reports, with a mean age of 11 years old. The rate of retinal detachment and joint problems is, therefore, very likely to be an underestimate, as these problems may develop in the future. It will be important to follow up these patients as the decades pass to better understand the lifetime risk of these problems.

## Figures and Tables

**Table 1:** Clinical features of patients with recessive pathogenic variants in *COL9A1*. All variants mentioned are homozygous in the affected patient.

Patient	Age	Gene	Variant	Refraction: RE/LE (D)	Retina	Vitreous	Face	Palate	Hearing	Joints	XR
1-I [11]	20 y	*COL9A1*	p.(Arg295Ter)	−5.5/−6	Normal	Abnormal	Normal	Normal	HFSNHL	Painful knees	Dysplasia of the femoral head with broadening of the femoral neck
1-II [11]	13 y	*COL9A1*	p.(Arg295Ter)	−16.75/−16.25	Perivascular lattice	Abnormal	Normal	Normal	HFSNHL	Hypermobility	Normal
1-III [11]	10 y	*COL9A1*	p.(Arg295Ter)	−6/−7.25	Atrophic retinal holes	Abnormal	Normal	Normal	HFSNHL	No symptoms	Maxillary hypoplasia, irregular femoral epiphyses
1-IV [11]	7 y	*COL9A1*	p.(Arg295Ter)	−5/−5	Normal	Abnormal	Normal	Normal	HFSNHL	No symptoms	Maxillary hypoplasia, irregular femoral epiphyses
2-I [12]	43 y	*COL9A1*	c.1519C > T, p.(Arg507Ter)	−6.25/−8	Progressive chorioretinal degeneration	Abnormal	Normal	-	HFSNHL	Spondylodesis due to thoracolumbar scoliosis	Short, broad femoral neck
2-II [12]	29 y	*COL9A1*	c.1519C > T, p.(Arg507Ter)	−6.5/−5.75	Epiretinal membrane, coats like vasculopathy. progressive chorioretinal degeneration	Abnormal	Normal	-	HFSNHL	No symptoms	Broad femoral neck
3-I [12]	15 y	*COL9A1*	c.883 > T, p.(Arg295Ter)	−18.5/−17	Bilateral retinal detachment	-	Flat midface, bilateral epicanthal folds, malocclusion with leftwards chin deviation	-	HFSNHL	No symptoms	Short and flat femoral neck, irregular femoral epiphyses, small squat metacarpals, small squat iliac wing
4-I [13]	6 y	*COL9A1*	c.1519C > T, p.(Arg507Ter)	−8.5/−7	Normal	Abnormal	Normal	Normal	HFSNHL	No symptoms	Normal

HFSNHL = high-frequency sensorineural hearing loss.

**Table 2:** Clinical features of patients with recessive pathogenic variants in *COL9A2*. All variants mentioned are homozygous in the affected patient.

Patient	Age	Gene	Variant	Refraction: RE/LE (D)	Retina	Vitreous	Face	Palate	Hearing	Joints	XR
5-I [14]	9 y	*COL9A2*	c.843_c.846 + 4del 8, p.(Asp281GlnfsTer70)	High myope	Lattice degeneration	Abnormal	Flat face	Normal	SNHL	No symptoms	Normal
5-II [14]	18 m	*COL9A2*	c.843_c.846 + 4del 8, p.(Asp281GlnfsTer70)	High myope	Not commented	-	Flat face, small mandible	Normal	SNHL	No symptoms	Not commented
6-I [13]	2 y	*COL9A2*	c.98delG, p.(Gly33AlafsTer53)	−9/−9	Normal	Abnormal	Flat midface	Normal	HFSNHL	No symptoms	Normal
7-I [13]	15 y	*COL9A2*	c.1692_1717del p.(Gly565TrpfsTer32)	−6.5/−7	Snailtrack degeneration. Flat round holes	Abnormal	Normal	Normal	HFSNHL	Mild hip pain	Refused by family
7-II [13]	9 y	*COL9A2*	c.1692_1717del p.(Gly565TrpfsTer32)	−4.5/−3	Lattice degeneration	Abnormal	Normal	Normal	HFSNHL	Nonspecific mild joint pain	Refused by family
8-I [15]	16 y	*COL9A2*	c.1332del p.(Val446Trpfs*85)	−1/−4	Unilateral retinal detachment	Abnormal	Subtle flat midface	Normal	SNHL	No symptoms	Not commented
8-II [15]	14 y	*COL9A2*	c.1332del p.(Val446Trpfs*85)	−7/−6	Normal	Abnormal	Subtle flat midface	Normal	SNHL	Plano valgus	Not commented

SNHL = sensorineural hearing loss (frequency not specified).

**Table 3:** Clinical features of patients with recessive pathogenic variants in *COL9A3*. All variants mentioned are homozygous in the affected patient unless stated otherwise.

Patient	Age	Gene	Variant	Refraction: RE/LE (D)	Retina	Vitreous	Face	Palate	Hearing	Joints	XR
9-I [16]	16 y	*COL9A3*	c.1176_1198del, p.(Gln393CysfsTer25)	−6	Normal	Normal	Normal	Normal	HFSNHL	No symptoms	Non-specific metacarpal and femoral epiphyseal abnormalities.
9-II [16]	11 y	*COL9A3*	c.1176_1198del, p.(Gln393CysfsTer25)	−8	Normal	Normal	Flat midface	Normal	HFSNHL	No symptoms	Non-specific metacarpal and femoral epiphyseal abnormalities.
9-III [16]	4 y	*COL9A3*	c.1176_1198del, p.(Gln393CysfsTer25)	−5	Normal	Normal	Flat midface	Normal	HFSNHL	No symptoms	Non-specific metacarpal and femoral epiphyseal abnormalities.
10-I [17]	12 y	*COL9A3*	c.650dupC	−10.75/−11.25	Not commented	Normal	Not commented	Normal	SNHL	No symptoms	Flattened proximal femoral epiphyses, mild platyspondyly
11-I [13]	20 y	*COL9A3*	c.1411C > T, (p.Arg471Ter)	−23/−23	Very myopic in appearance	Abnormal	Normal	Normal	HFSNHL	Severe arthropathy in shoulders and hip, mobilises in wheelchair	Spinal scoliosis surgery. Joint space narrowing both knees.
11-II [13]	18 y	*COL9A3*	c.1411C > T, p.(Arg471Ter)	−7/−7	Unilateral retinal detachment	Abnormal	Normal	Normal	HFSNHL	No symptoms	Normal
12-I [18]	2 y	*COL9A3*	Compound heterozygous c.268C > T p.(Arg90Ter) AND c.1729C > T p.(Arg577Ter)	High myopia	Peripheral pigmentation	-	Flat midface	High	SNHL	Lower limb joint pain with valgus knees	Several, including: Spondylepiphyseal dysplasia. Spina bifida occulta, mild platyspondyly, irregular femoral heads, flattened distal femoral epiphyses

**Table 4:** Clinical features of patients with recessive pathogenic variants in *COL11A1*. Variants are specified as homozygous or compound heterozygous.

Patient	Age	Gene	Variant	Refraction: RE/LE (D)	Retina	Vitreous	Face	Palate	Hearing	Joints	XR
13-I [19]	6 y	*COL11A1*	Homozygous c.1191delT, p.(Asn398Metfs*19)	-	Unilateral retinal detachment	-	Flat midface, micrognathia	Cleft palate	SNHL	-	-
14-I [20]	Adult	*COL11A1*	Compound heterozygous c.1191delT, p.Asn398Metfs*19 AND c.4259G > T, p.Gly1420Val	+3.5/+4	-	Abnormal	-	Cleft palate	SNHL	-	-
15-I [20]	20 m	*COL11A1*	Compound heterozygous c.1421dupC, p.Gly475Argfs*9 AND c.991-24A > G	−7/−7	-	-	Pierre Robin sequence	-	SNHL	-	-
15-II [20]	18 d	*COL11A1*	Compound heterozygous c.1421dupC, p.Gly475Argfs*9 AND c.991-24A > G	-	-	-	Pierre Robin sequence	-	SNHL	-	-
16-I [20]	2 y	*COL11A1*	Compound heterozygous c.2607A > G, p.Ala869Ala AND c.5398G > T, p.Gly1800Cys	−12.5/−12.5	-	Abnormal	-	Normal	SNHL	Laxity	Normal
17-I [21]	3 y	*COL11A1*	Homozygous c.1168G > T, p.(Glu390Ter)	−8.5/−9	-	Normal	-	-	SNHL	Normal	Normal
18-I [22]	16 y	*COL11A1*	Compound heterozygous c.1245 + 2T > C AND c.4109_4126del, p.Ala1370_Gly1375del	−10/−5	Retinal atrophy, retinal tears	Posterior vitreous detachment	-	Normal	SNHL	Normal	Normal

**Table 5:** Clinical features of patients with pathogenic variants in *LRP2* and *LOXL3.* All variants mentioned are homozygous in the affected patient.

Patient	Age	Gene	Variant	Refraction: RE/LE (D)	Retina	Vitreous	Face	Palate	Hearing	Joints	XR
19-I [23]	14 y	*LRP2*	c.11483A > G, p.(Asp3828Gly)	−22/−22	Myopic	Abnormal	Flat midface, retrognathia	-	Normal	Normal	Normal
19-II [23]	8 y	*LRP2*	c.11483A > G, p.(Asp3828Gly)	−20/−20	Unilateral giant retinal tear detachment	Abnormal	Flat midface	-	Normal	Upper and lower limb joint pain	Normal
20-I [24]	16 y	*LOXL3*	c.2027G > A, p.(Cys676Tyr)	−10/−10	Lattice degeneration	-	Flat midface, micrognathia	Cleft palate	Mild conductive loss	Normal	Normal
20-II [24]	8 y	*LOXL3*	c.2027G > A, p.(Cys676Tyr)	−8/−8	Lattice degeneration	-	Flat midface, micrognathia	Cleft palate	Normal	Normal	Normal
21-I [25]	11 y	*LOXL3*	c.1036C > T, p.(Arg346Trp)	−7.5/−8	Myopic	Posterior vitreous detachment	Normal	High arched	-	Laxity	Normal
21-II [25]	40 y	*LOXL3*	c.1036C > T, p.(Arg346Trp)	Unknown [LASIK]	Myopic	-	Normal	-	-	Normal	Normal
22-I [26]	16 y	*GZF1*	c.865G > T, (p.Glu289Ter)	High myopia	-	-	-	-	Hearing loss	Hypermobility, joint dislocations, severe scoliosis, talipes	Kyphosis, scoliosis, talipes
22-II [26]	2.5 y	*GZF1*	c.865G > T, (p.Glu289Ter)	High myopia	-	-		-	Normal	Hypermobility, joint dislocations, talipes	Scoliosis, talipes
23-I [26]	20 y	*GZF1*	c.1054dup, p.(Thr352Asnfs*50)	High myopia	Retinal detachment, chorioretinal coloboma	-	-	-	Hearing loss	Hypermobility, scoliosis	Scoliosis
23-II [26]	16 y	*GZF1*	c.1054dup, p.(Thr352Asnfs*50)	High myopia	Retinal detachment, chorioretinal coloboma	-	-	-	Normal	Hypermobility, talipes	-
23-III [26]	7 y	*GZF1*	c.1054dup, p.(Thr352Asnfs*50)	High myopia	-	-	-	-	Normal	Hypermobility	-

**Table 6:** Comparison of prevalence of clinical features of dominant vs. recessive SS.

Clinical Feature	Dominant SS	Recessive SS
Myopia (%)	80	98
Retinal detachment (%)	42–62	18
Palate anomalies (%)	28–45	10
Hearing impairment (%)	50–75	78
Joint pain (%)	41–90	15
